# Supplementary material for: Waveguide-coupled nanopillar metal-cavity light-emitting diodes on silicon
Source: Nat Commun. 2017 Feb 2;8:14323. doi: 10.1038/ncomms14323 (PMC5296653; doi:10.1038/ncomms14323)
Supplement: Supplementary Information — Supplementary Figures, Supplementary Notes, Supplementary Tables and Supplementary References. [file ncomms14323-s1.pdf]

Supplementary Note 1: Modeling of grating coupler

For characterization purposes, the nanopillar LED is connected to a grating coupler by means of an adiabatic taper. The grating coupler was modeled with two-dimensional finite-difference time-domain (FDTD) simulations as described in the Methods section of the main text. Supplementary Figure 1: shows the calculated grating coupler performance as a function of wavelength. The left axis corresponds to the chip-to-free space outcoupling efficiency, i.e. power diffracted upwards into the first diffraction order normalized by the power in the transverse-electric mode injected to the grating coupler through the waveguide. The right axis shows the diffraction angle at which the light is diffracted. This chip to free-space outcoupling is performed at diffraction angles below 24° for the wavelength range 1.4 – 1.6 μm, which is within the numerical aperture (NA = 0.42) used for the light collection during the characterization.

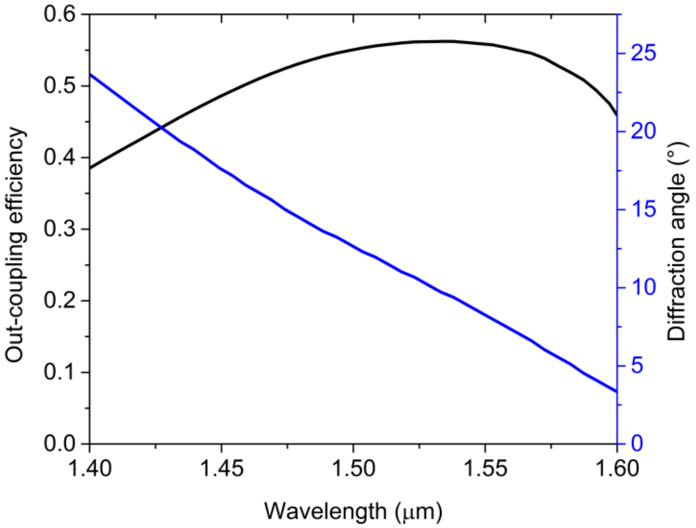

Supplementary Figure 1: Grating coupler performance. The coupler shows an efficiency of 55% at 1.55 μm. Left: chip-to-free space coupling efficiency. Right: angle at which the intensity of the diffracted optical distribution is maximum.

Supplementary Note 2: Device fabrication

The fabrication process required a variety of techniques, such as optical and electron beam lithography (EBL), plasma-enhanced chemical vapor deposition (PECVD) of dielectrics, reactive ion etching (RIE) processes, wet-chemical etching, thermal and electron-beam evaporation of metals, rapid thermal annealing, etc. The definition of the pillar, waveguide and grating coupler is carried out by EBL in three different steps and the rest of the process is done by optical lithography. EBL was used in order to circumvent the limited resolution of optical lithography

systems in our facilities, but our nano-LED devices could be developed using other lithographic methods widely employed in photonics, namely deep UV lithography [1].

An overview of the fabrication process flow to fabricate metal-coated nanopillars on top of waveguides, which are connected to grating couplers, in III-V membranes bonded to a silicon substrate is presented in Supplementary Figure 2:. The use of the benzocyclobutene (BCB) [2] bonding layer allows an InP-waveguide with high refractive index contrast (resulting in a compact cross section) which facilitates the coupling with the nanocavity mode with high confinement.

In the following, the process flow is described in 10 stages which are depicted in Supplementary Figure 2:, and supported by experimental results shown in Supplementary Figure 3: and Supplementary Figure 4:.

1. At first, the III-V layer stack is epitaxially grown on an InP substrate by metal-organic chemical vapor deposition (MOCVD) and adhesively bonded to a silicon substrate with BCB. After bonding, the InP substrate is selectively removed by wet etching using H_2O:HCl (1:4).
2. The definition of the pillar is done using hydrogen silsesquioxane (HSQ) negative resist, which is well known for its usage in high resolution lithography [3], in combination with HPR504 to form a bilayer resist scheme for etching structures with high aspect ratio (i.e. the nanopillar) [4]. First, a SiO_2 (450nm) hardmask is deposited with PECVD, and then HPR540 (450 nm) and HSQ (100 nm) resists are spun. After e-beam lithography, HSQ

development is done with MaD531S and the pattern is transferred to the HPR layer with an O_2-plasma reactive-ion etching (RIE). Then, the pattern is transferred to SiO_2 with a CHF_3 chemistry, and finally the semiconductor nanopillar is etched with an inductively coupled plasma (ICP) RIE process based on methane-hydrogen (CH_4:H_2). The ICP-RIE typically provides higher etching anisotropy that RIE, which translates into more vertical sidewalls. The result is shown in Supplementary Figure 3:a.

3. Later, a new HSQ resist layer is spun while keeping the previous SiO_2 on top of the nanopillar and e-beam patterned as shown in Supplementary Figure 3:b, using again MaD531S as developer. The HSQ selectivity against semiconductor etching is enhanced by treating it with an oxygen plasma [5]. Then, the HSQ resist pattern is used as hardmask to dry etch the waveguide while protecting the pillar with its original SiO_2 hardmask. Supplementary Figure 3:c shows the result after removal of the masking layers.
4. An optical lithography is performed using AZ4533 resist and MaD531S developer (see Supplementary Figure 4:a), in order to expose the waveguides and wet etch their top p-doped InGaAsP cladding with H_2O:H_2SO_4:H_2O_2 (10:1:1), which otherwise would cause strong waveguide loss due to intraband absorption at 1.55 μm. Supplementary Figure 4:b shows the result.
5. The next step is to fabricate the grating coupler. For this, a Si_3N_4 layer is deposited with PECVD and then ZEP520A resist is spun. The resist is e-beam patterned and developed with n-Amyl Acetaat; then, the pattern is transferred into the Si_3N_4 hardmask with a CHF_3 based RIE.

6. In order to protect the nanopillar during the grating etching into the semiconductor, an optical lithography is carried out using again an AZ4533 resist and the MaD531S developer (see Supplementary Figure 4:c). The pattern is hardbaked to withstand a CH_4:H_2 RIE etch with which the grating coupler is etched into the InP layer. The result is shown in Supplementary Figure 4:d after resist and hardmask removal.
7. Once the nanopillar, waveguide and grating coupler are fabricated, a SiO_2 layer (175 nm) is conformally deposited with PECVD as shown in Supplementary Figure 3:d, to serve as dielectric cladding for the cavity and for electrical insulation of the diode structure. Then, Au/Ti (40 nm/50 nm) adhesion pads are deposited around the pillar with a lift-off process, whose purpose is to promote the adhesion of silver later on (see Supplementary Figure 4:e and Supplementary Figure 4:f).
8. In order to contact electrically the n-InGaAs layer, the SiO_2 must be removed from the pillar top. For this, MaN440 resist (thicker than the nanopillar height) is spun on the chip, cured (95°C, 5min) and slowly developed (etched back) with an MaD531S:H_2O (2:1) solution at a rate of 7 nm/s, until the top of the nanopillars is exposed. Then, the SiO_2 is etched only from the nanopillar top with RIE (CHF_3:O_2). The result is shown in Supplementary Figure 3:e after resist removal.
9. The next step consists of depositing metals to form the metal-cavity as well as the n-contact. For this, Ag/Ge (>300 nm/4 nm) layers are deposited all over the wafer sample by thermal evaporation and treated with rapid thermal annealing (RTA) at 350 °C for 30 seconds to (1) provide a low contact resistance n-type ohmic contact and (2) to increase

the silver grain size to reduce its optical loss (see Supplementary Figure 3:f). Afterwards, a 100 nm thick Au layer is sputtered to prevent silver oxidation. Then, an optical lithography is performed with AZ5433 resist and MaD531S developer in order to protect the n-contact pad and the nanopillar regions (see Supplementary Figure 4:g) during wet etching of the metal layers Au/Ag with a KCN-based solution. Supplementary Figure 4:h shows the result after the photoresist has been removed.

10. Finally, a last optical lithography step is required to fabricate the p-contact pad with lift-off using MaN440 resist and MaD531S developer (see Supplementary Figure 4:i and Supplementary Figure 4:j). For this, firstly the SiO_2 is removed with buffered hydrofluoric etch (BHF) to have access to the p-InGaAsP contact layer and then a standard Au/Pt/Ti (200 nm/75 nm/60 nm) metallisation is performed.

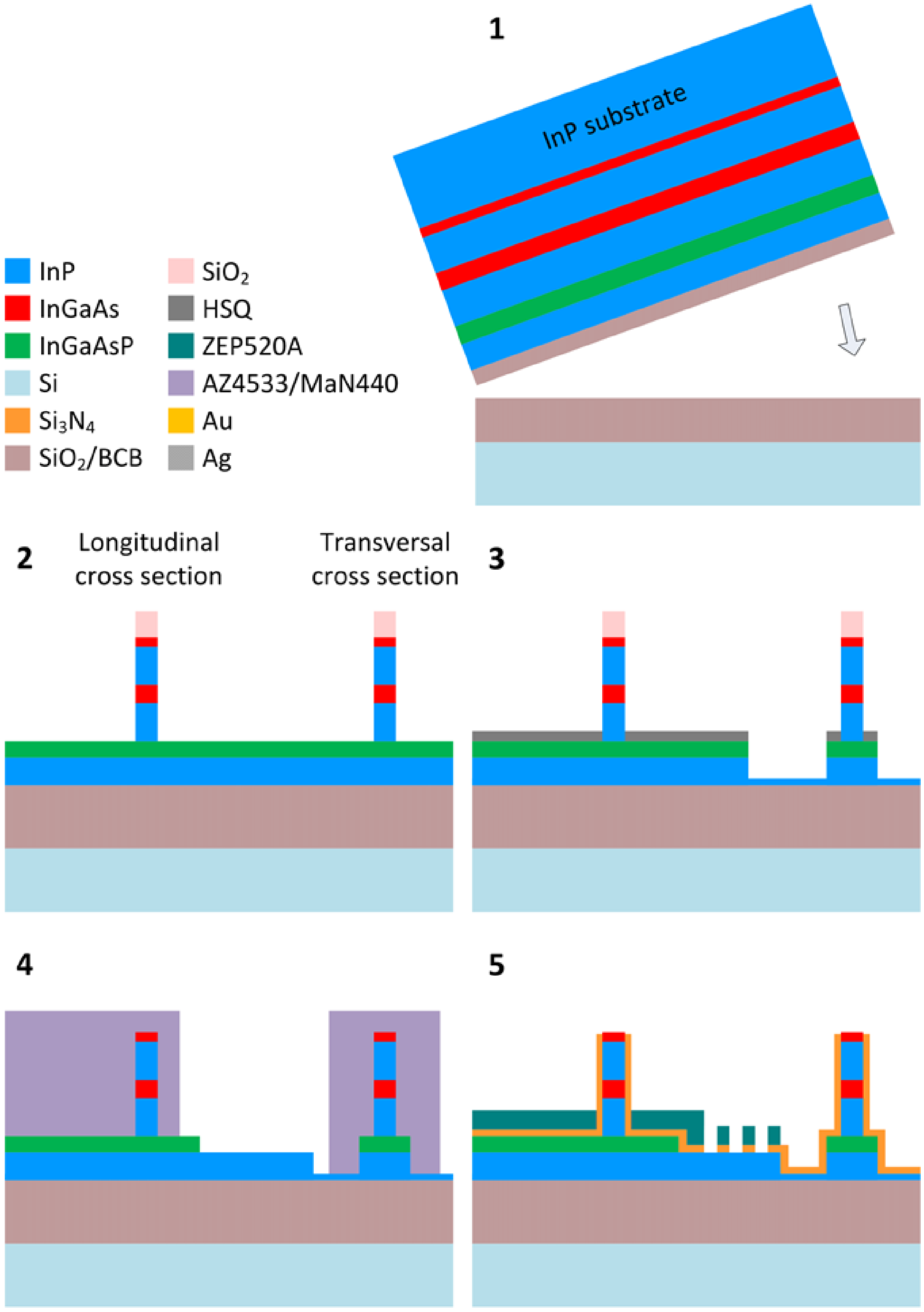
1
InP substrate
InP
InGaAs
InGaAsP
Si
Si3N4
SiO2/BCB
SiO2
HSQ
ZEP520A
AZ4533/MaN440
Au
Ag
2
Longitudinal cross section
Transversal cross section
3
4
5

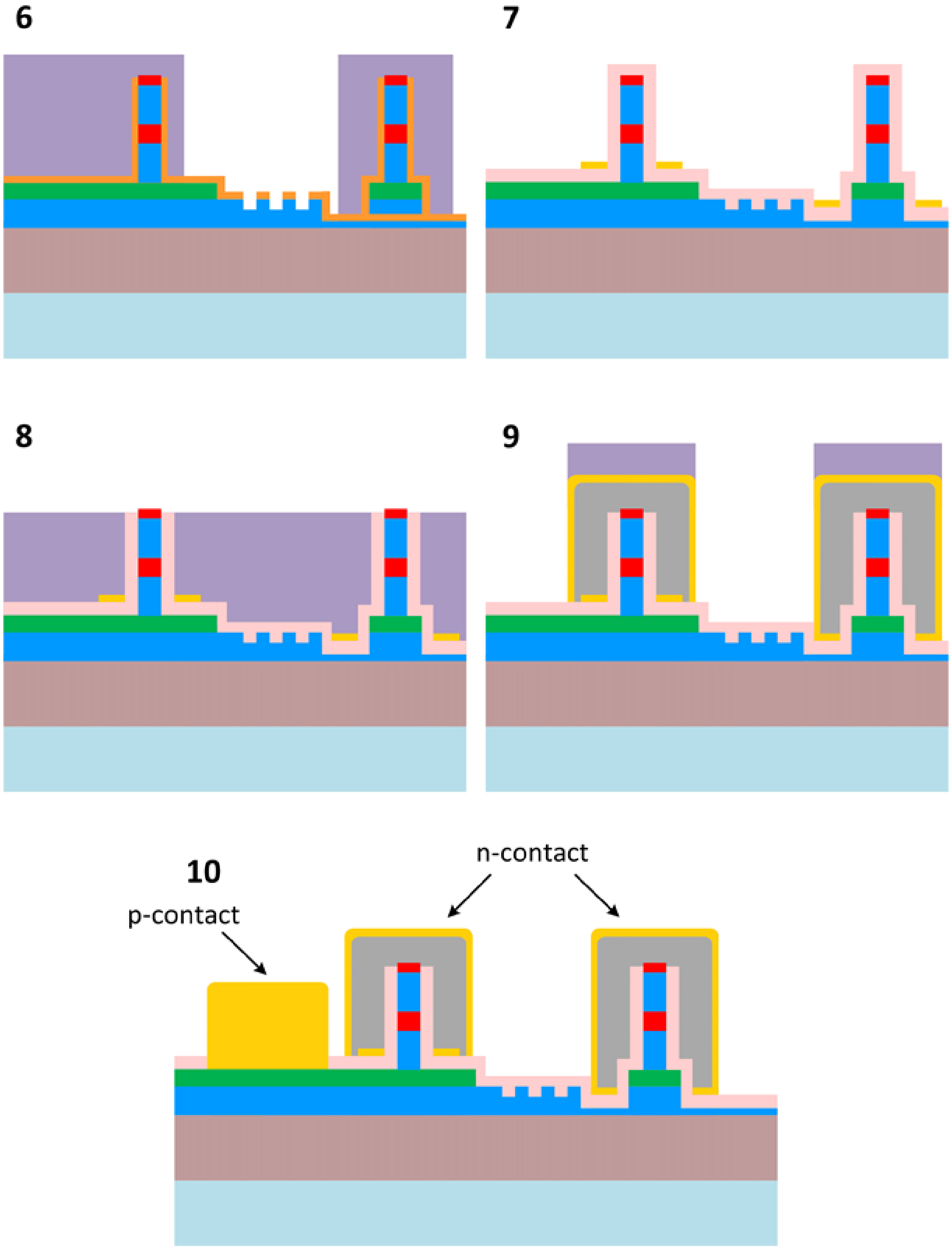

Supplementary Figure 2: Process flow to fabricate metal-cavity nanopillars coupled to waveguides in III-V membranes on silicon. The longitudinal and transversal cross sections are shown (i.e. along and perpendicular to the waveguide, respectively).

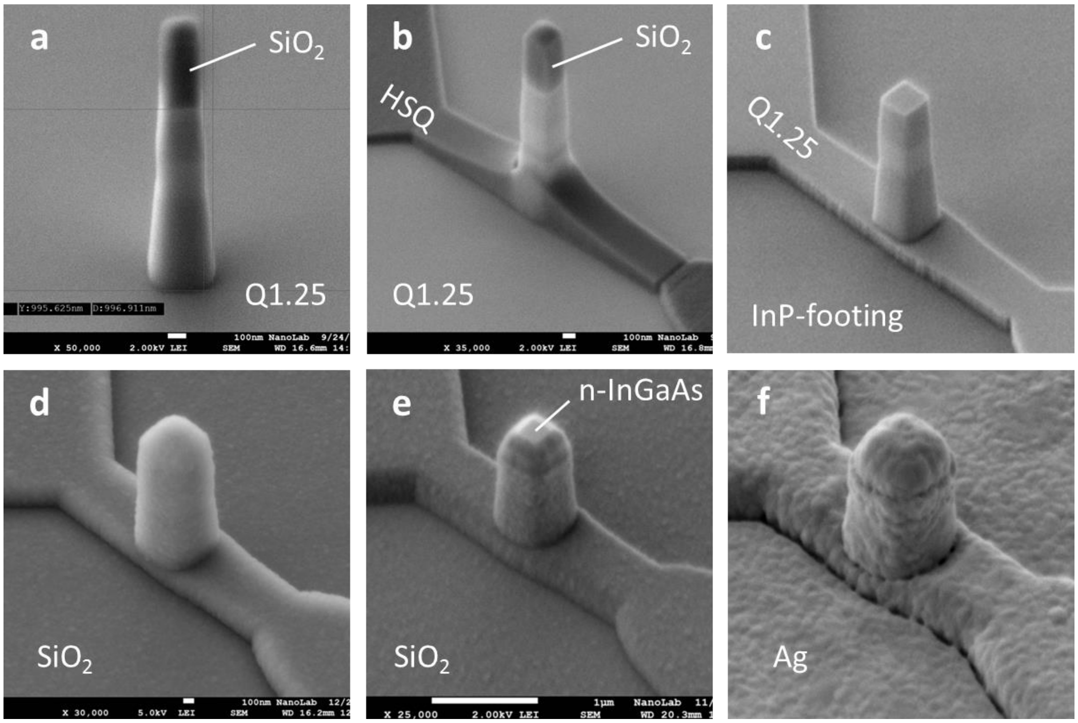

Supplementary Figure 3: Scanning electron microscope images showing the fabrication process with focus on the nanopillar. (a) Image after the etching of the nanopillar. (b) After the development of HSQ resist to act as waveguide hardmask. (c) After waveguide etching and removal of hardmask layers. (c) After waveguide etching and removal of masking layers. (d) After deposition of SiO_2 cladding. (e) After etching of SiO_2 from the pillar top to expose the n-doped InGaAs contact layer. (d) After deposition of Ag/Ge and rapid thermal annealing.

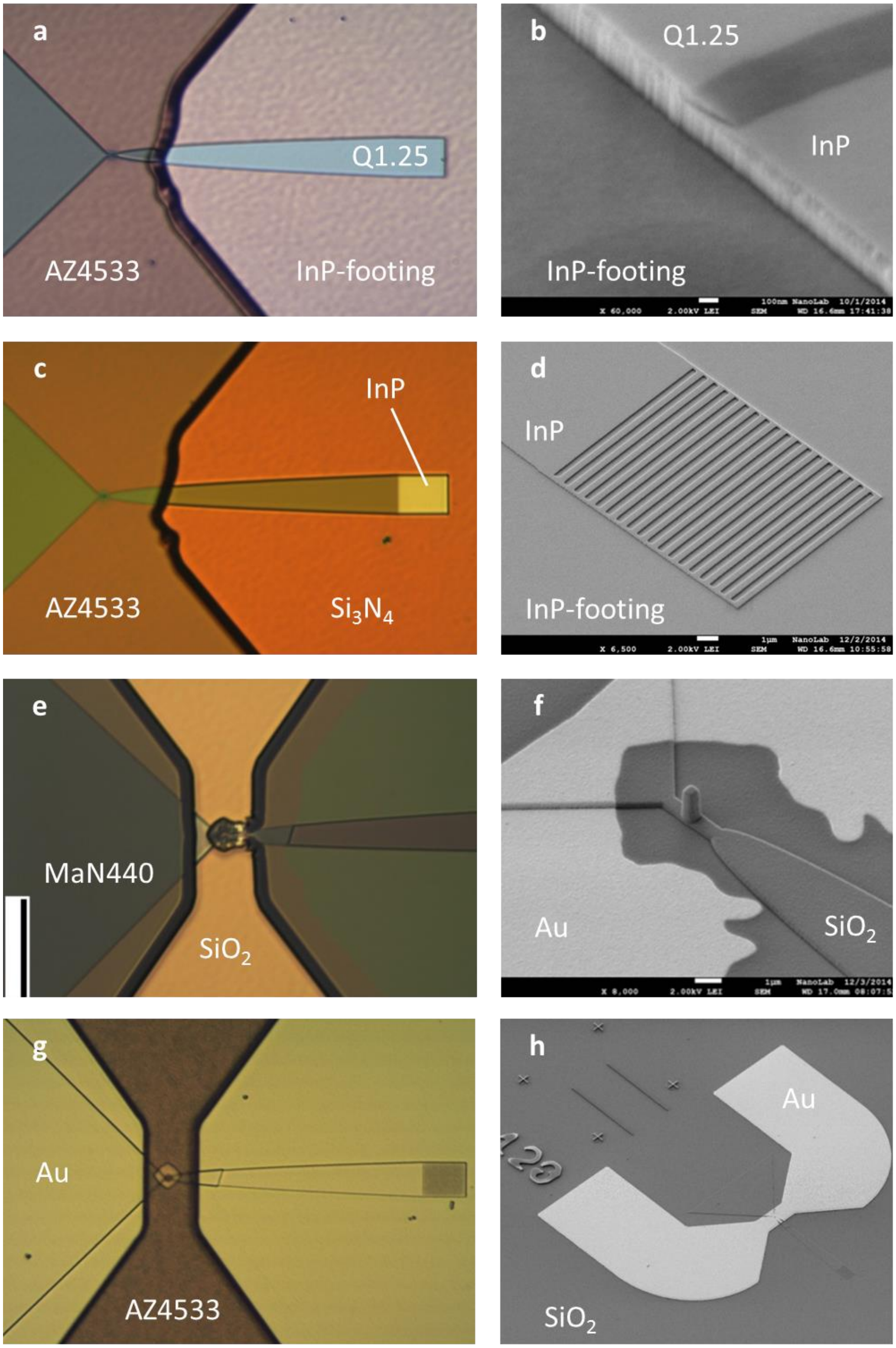
a
Q1.25
AZ4533
InP-footing
b
Q1.25
InP
InP-footing
c
InP
AZ4533
Si3N4
d
InP
InP-footing
e
MaN440
SiO2
f
Au
SiO2
g
Au
AZ4533
h
Au
SiO2

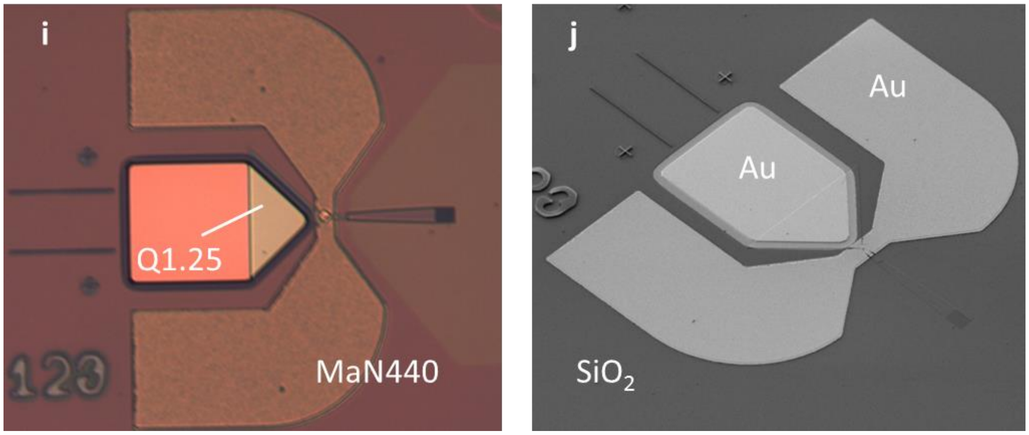

Supplementary Figure 4: Microscope photos showing the fabrication process with focus on the large scale patterns. (a) Sample after optical lithography and development to expose the Q1.25 cladding on top of waveguides. (b) Waveguide edge after wet etching of Q1.25 from the top of the InP-waveguide and removal of AZ4533. (c) Sample with the grating pattern defined in a Si_3N_4 layer while protecting the pillar with AZ4533 resist. (d) Sample after dry etching of the grating coupler into the semiconductor and removal of the AZ4533 protection resist and Si_3N_4 hardmask. (e) Sample after optical lithography and development of MaN440 resist to deposit the adhesion layers Au/Ti. (f) Sample after deposition of Au/Ti adhesion layers by lift-off around the nanopillar. (g) Sample after optical lithography and development of AZ4533 resist to protect the pillar and n-contact region in order to wet etch the Au/Ag layers. (h) Sample after etching the metal layers Au/Ag everywhere except in the device region and removal of photoresist. (i) Sample after optical lithography and wet etch of SiO_2 cladding to access the Q1.25 layer in order to do the p-contact metallization. (j) Sample after deposition of Au/Pt/Ti metallization layers by lift-off.

Supplementary Note 3: Time-resolved experiments on nanopillars

In order to confirm that non-radiative recombination is indeed the main process involved in the sub-nanosecond carrier lifetimes shown in Fig. 4a, we performed a systematic study of the carrier dynamics in single InP-InGaAs-InP nanopillars with varying cross section, using micro photoluminescence (PL) spectroscopy and time-resolved PL spectroscopy. Supplementary Figure 5:b shows a scanning electron microscope (SEM) picture of a typical fabricated nanopillar including the epilayer structure. The nanopillars were covered by a 175 nm thick layer

of insulator SiO_2 similarly as used in the nanopillar LEDs. Since these nanopillars were not covered by the metal cladding, the measurements could directly probe optically the free carrier dynamics allowing a comprehensive analysis of the optical quality of the InGaAs surface in terms of carrier lifetimes.

The time-resolved photoluminescence (TRPL) curves, presented in Supplementary Figure 5:a for different nanopillar side lengths, indicate minority carrier lifetimes in the nanopillars in the order of hundreds of picosecond for pillars with sizes similar to the nanopillar LEDs, which are much shorter than the nanosecond-range lifetime of the bulk and compare with the sub-nanosecond response measured in Fig. 4a. On the basis of the measured lifetimes determined by the TRPL measurements, the surface recombination velocity υ_s can be estimated by the equation

$$\frac{1}{\tau} = \frac{1}{\tau_b} + \frac{4\upsilon_s}{d} \qquad (1)$$

where τ is the effective carrier lifetime, τ_b is the carrier lifetime in the bulk material, and d is the side length of the pillar with square cross section. From the fitting with Eq.1, we estimate a surface recombination velocity of around 5×10^4 cm s^{-1} for the pillars with cross section area below 0.5 μm^2. The estimated values are nearly four times higher than the measured values of InGaAs lattice-matched to InP with improved passivation techniques [6], indicating that the surface of the nanopillars has been affected by the process.

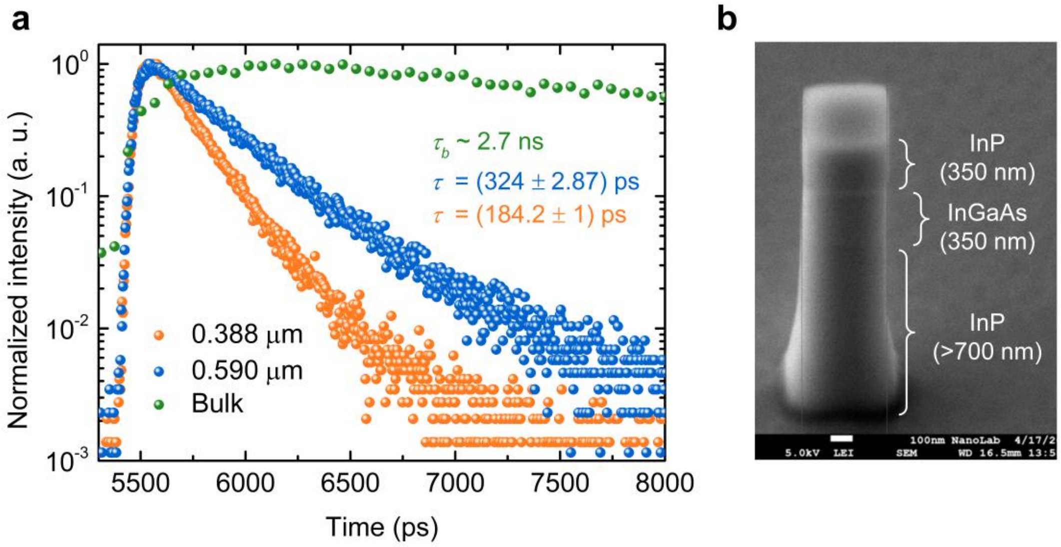

Supplementary Figure 5: Time-resolved photoluminescence measurements of nanopillars. (a) Comparison between photoluminescence signal of nanopillars and bulk InGaAs. The legend indicates the corresponding nanopillar side length. (b) Typical nanopillar fabricated for the photoluminescence experiment.

Supplementary Note 4: Experimental setup

The dynamic characterization of the nanopillar LEDs was carried out with the setup depicted in Supplementary Figure 6: as described in detail in the Methods section of the main text. The devices were probed electrically through a bias-T to provide both a D.C. bias and an R.F. signal containing the modulation pattern. The optical signal emitted by the LED was collected by a microscope objective from the devices' integrated grating coupler, coupled to an optical fiber and filtered (FWHM~50 nm centered at 1550 nm). The optical signal is then detected with a superconducting single photon detector (SPD). A correlation card (Pico Harp 300) is used to

measure the time delay between the excitation signal (trigger) and the optical signal (stop). A histogram of these arrival times corresponds to the time-dependent electro-optical modulation.

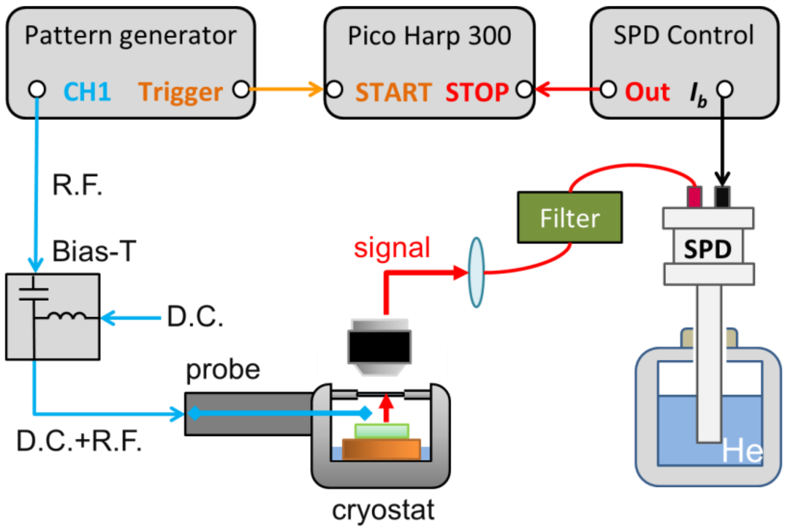

Supplementary Figure 6: Schematic of time-resolved electroluminescence setup. The setup was employed to characterize the dynamic response of the nanopillar LEDs.

Supplementary Note 5: Nano-LED L-I characteristics

As the cavity resonance frequency is very sensitive to nanopillar size, we fabricated a series of devices with different symmetric cross sections ranging between 300 nm × 300 nm and 400 nm × 400 nm. The tested nanocavities (we tested devices ranging from 300 nm × 300 nm to 350 nm × 350 nm) with identical horizontal cross sections displayed similar performances in terms of optical output and maximum external quantum efficiencies indicating a good yield. In

Supplementary Fig. 7, we present a selection of additional measured L-I characteristics at low-temperature.

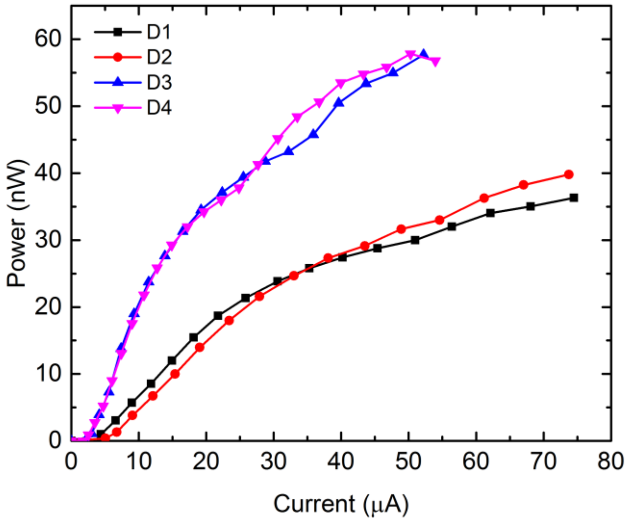

Supplementary Figure 7: Light-current characteristics of metal-cavity nano-LED devices. The devices (D1-D4) are measured at low-temperature (9.5 K) and have identical horizontal cross sections of D1-D2: 340 nm×340 nm; D3-D4: 350 nm×350 nm.

Supplementary Note 6: Operating energy versus injected current

Supplementary Fig. 8 shows the operating energy versus injected current (measurements at room temperature of the device measured in Fig. 4). Note that during the modulation speed measurements the nano-LED was operated at moderate and low bias pumping conditions (bias current <40 μA), mainly to avoid devices' thermal heating and possible failure, displaying with these operating conditions energy consumptions below 100 fJ/bit. The estimated energy

consumption when the nano-LEDs are operated at around the maximum output optical power (bias current of 100 μA) is ~540 fJ/bit.

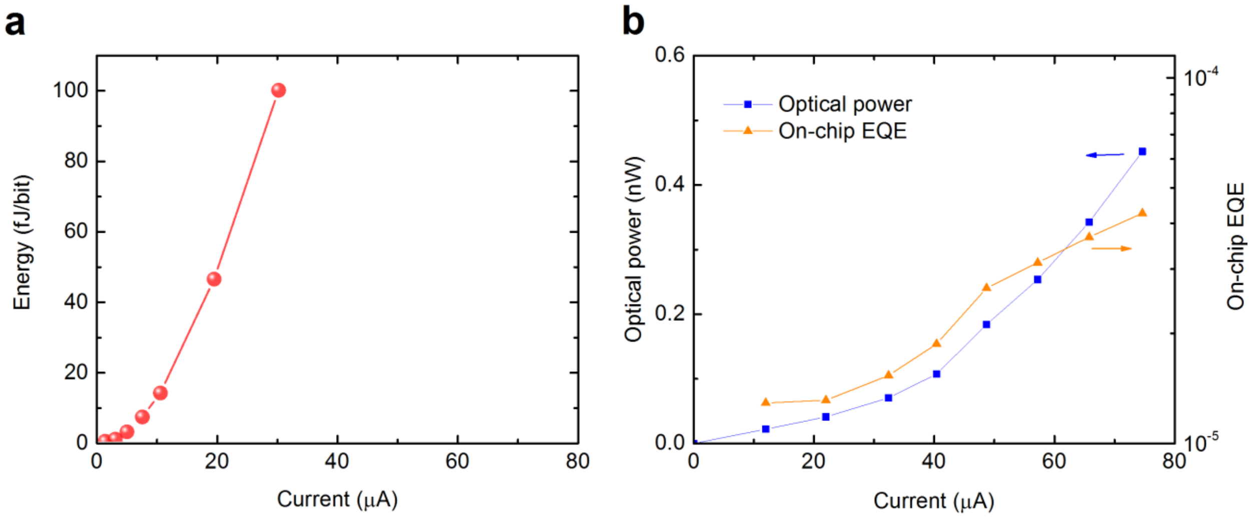

Supplementary Figure 8: Energy consumption of nano-LED devices. (a) Operating energy versus injected current at room temperature. The operating energy is calculated as the product of the injected current and applied voltage divided by 1.3 times the 3 dB bandwidth extracted from the fitted lifetime measured from the time-resolved electroluminescence (as measured in Fig. 4). (b) Optical output power and on-chip EQE versus injected current. The on-chip EQE is calculated as in the inset of Fig. 3b.

Supplementary Note 7: Purcell enhancement of the spontaneous emission

Spontaneous emission from Fermi's golden rule

The rate of photon emission for a homogeneously broadened emitter in a resonant cavity is derived directly from Fermi's golden rule[7]:

$$R_{\rm em} = \frac{2\pi}{\hbar^2}\int_0^{\infty} \left|\langle f|H|i\rangle\right|^2 \rho(\omega) L(\omega)\, d\omega \qquad (2)$$

where $L(\omega)$ is the homogeneous broadening lineshape, $\rho(\omega)$ the density of photon states, H the dipole-field interaction Hamiltonian and i,f the initial and final states of the dipole transition. The lineshapes for the cavity and the emitter are both typically given by Lorentzians.

Considering an electric dipole transition and a quasi-single-mode emission, the matrix element for the spontaneous emission of a photon is given by:

$$\left|\langle f|H|i\rangle\right| = E_0(\vec{r}_{\mathrm{em}})\,\hat{e}\cdot\vec{d}_{\mathrm{if}} \tag{3}$$

where $E_0(\vec{r}_{\mathrm{em}}) = \sqrt{(\hbar\omega/2\varepsilon_0\varepsilon_{\mathrm{ra}}V)}\,e(\vec{r}_{\mathrm{em}})$ is the magnitude of the field per photon at the position of the emitter $\vec{r}_{\mathrm{em}}$, $\hat{e}$ is a unit vector indicating its polarisation, ε_0 is the dielectric permeability of free space, $\varepsilon_{\mathrm{ra}}$ is the relative dielectric constant in the active material and V the effective cavity volume. The adimensional mode function $e(\vec{r}_{\mathrm{em}})$ is normalized to be $\left|e(\vec{r}_{\mathrm{em}})\right|_{\max} = 1$, so that for a point-like, optimally positioned emitter $\left|\langle f|H|i\rangle\right| = \sqrt{(\hbar\omega/2\varepsilon_0\varepsilon_{\mathrm{ra}}V)}\,\hat{e}\cdot\vec{d}_{\mathrm{if}}$, in agreement with the usual notation [8]. The atomic dipole moment is defined as:

$$\vec{d}_{\mathrm{if}} = \left|\langle\Psi_{\mathrm{l}}|\vec{d}|\Psi_{\mathrm{u}}\rangle\right| \tag{4}$$

where $\vec{d}$ is the dipole operator, and $\Psi_{\mathrm{u(l)}}$ is the upper (lower) level wave function of the atom.

In the case of a dielectric cavity, V is given by the energy normalization condition

$$\int 2\varepsilon(\vec{r})\left|E_0(\vec{r})\right|^2 d^3\vec{r} \equiv \frac{\hbar\omega}{\varepsilon_0\varepsilon_{\mathrm{ra}}V}\int \varepsilon_0\varepsilon_{\mathrm{r}}(\vec{r})\left|e(\vec{r})\right|^2 d^3\vec{r} = \hbar\omega \tag{5}$$

$$V = \int \frac{\varepsilon_{\mathrm{r}}(\vec{r})}{\varepsilon_{\mathrm{ra}}}\left|e(\vec{r})\right|^2 d^3\vec{r} \tag{6}$$

In the case where the dielectric constant is uniform in the cavity, the normalization condition simplifies to $V = \int \left| e\left(\vec{r}\right) \right|^2 d^3\vec{r}$ and the effective mode volume is close to the physical cavity volume. Note that the normalization condition, Eq. (6), changes in the case of a cavity with metal boundaries, since in that case the energy in the field and the kinetic energy of the electrons have to be both properly accounted for, as thoroughly discussed in the work of Khurgin and Sun [9].

From Eq. (2), the spontaneous emission rate of an emitter placed at the peak of the electric field, spectrally narrower than the cavity and resonant with it, can be derived as a special case. The ratio of this cavity spontaneous emission rate to the one in the bulk can be written as the well-known Purcell factor, $F_{\mathrm{p}} = (3\lambda^3 Q)/(4\pi^2 V)$, where λ is the wavelength associated with the transition and Q is the quality factor of the cavity mode. The Purcell factor, F_{p}, is valid only if the cavity's resonance linewidth is larger than the emission linewidth of the emitter, which would for example correspond to a single quantum-dot nanocavity LED or laser at low temperature, a case investigated in [10]. However, this limit does not apply to our nano-LED devices employing a broad gain active medium (bulk InGaAs) and operating at room temperature. Using Eq. (2) in the case of a single optimally-placed emitter with homogeneous broadening, $\Delta\omega_{\mathrm{em}} >> \Delta\omega_{\mathrm{cav}}$, the spontaneous emission rate in the cavity mode (approximating $\rho(\omega)$ by a Dirac delta funtion) becomes

$$R_{\mathrm{sp,cav}} \approx \frac{\pi}{\hbar\varepsilon_0\varepsilon_{\mathrm{ra}}} \frac{\omega_{\mathrm{cav}} d_{\mathrm{if}}^2}{\Delta\omega_{\mathrm{em}} V} \qquad (7)$$

where ω_{cav} is the cavity frequency. In Eq. (7) we verify that, apart from other parameters, only

the 1/*V* dependence appears in the spontaneous emission term, which comes directly from the dependence of the spontaneous emission rate on the vacuum field (also appearing in the Purcell factor equation).

In the case of a bulk semiconductor active medium, the inhomogeneous broadening of the electronic states must be described by integrating Eq. (2) over the bands. In the limit $\Delta\omega_{em} >> \Delta\omega_{cav}$, the spontaneous emission rate per unit time and volume, $r_{sp,cav}(n)$, becomes:

$$r_{sp,cav}(n) = \frac{\pi}{\hbar\varepsilon_0\varepsilon_{ra}}\frac{\omega_{cav}d_{if}^2}{V}\int_{\omega_g}^{\infty}\rho_j(\omega_{vc})L(\omega_{cav}-\omega_{vc})f_c\left[1-f_v\right]d\omega_{vc} \tag{8}$$

where $\rho_j(\omega_{vc})$ is the joint density of electronic states per unit frequency and volume, and f_c, f_v are the Fermi distribution functions of electrons in the conduction band and valence bands, respectively. The conduction Fermi functions are computed from the electron density, n_c, and hole density, n_h, related to the respective quasi-Fermi levels $E_{f,c}$ and $E_{f,v}$. Finally, in the calculations of $r_{sp,cav}(n)$ we assume the charge-neutrality condition ($n_c = n_h$).

Rate equations model

Using Eq. (8) describing the photon creation rate by spontaneous emission in a resonant cavity we can write the rate equations for carrier density *n*, and photon density, n_{ph}, employed to describe our electrically pumped nanopillar LED:

$$\frac{dn}{dt} = \frac{\eta_i I}{qV_a} - r_l(n) - r_{nr}(n) - \frac{V_{a,eff}}{V_a}r_{sp,cav}(n) \tag{9}$$

$$\frac{dn_{\mathrm{ph}}}{dt} = \frac{V_{\mathrm{a,eff}}}{V} r_{\mathrm{sp,cav}}(n) - \frac{n_{\mathrm{ph}}}{\tau_{\mathrm{p}}} \quad (10)$$

where I is the injection current, q is the electron charge, η_{i} the injection efficiency, and V_{a} is the volume of the active material. The rate of stimulated emission is assumed to be negligible and it is not included in the model. The rates describing the physical processes occurring in the nano-LED include the radiative decay rate into the cavity mode, Eq. (8), the non-radiative recombination rate, $r_{\mathrm{nr}}(n) = \frac{\upsilon_{\mathrm{s}} A}{V_{\mathrm{a}}} n + Cn^3$ (accounting for surface recombination, described by the surface velocity, υ_{s}, and by the surface area of the active region, A, and for Auger recombination, described by C), and the radiative decay rate into the leaky modes, $r_{\mathrm{l}}(n)$. This rate is calculated similarly as $r_{\mathrm{sp,cav}}(n)$ but replacing the cavity emission by the emission of a density of optical modes in the free space, $\alpha n_{\mathrm{ra}}^3 \omega^2 / \pi^2 c^3$, where n_{ra} is the refractive index and α is a fitting parameter describing the suppression of the emission of the leaky modes due to the presence of the metal nanocavity.

The prefactor $V_{\mathrm{a,eff}}$ in Eqs. (9) and (10) accounts for the spatial variation of the field in the active region of our nanocavity [see the calculated mode field profile of Fig. 1c)]. We discretize the active volume in boxes, dV_{a}, small enough that the field $e(\vec{r})$ is approximately uniform, and sufficiently large that a band description is adequate (size > 40 nm). Assuming that the carrier concentration is uniform over V_{a} due to fast carrier diffusion, each box contributes to a cavity emission rate $r_{\mathrm{sp,cav}}(n) dV_{\mathrm{a}}$, and the integration over V_{a} provides the effective active

volume $V_{\mathrm{a,eff}} = \int_{V_a} |e(\vec{r}_a)|^2 \, dV_a$.

Finally, the photon loss rate is given by $n_{\mathrm{ph}} / \tau_{\mathrm{p}}$, where $\tau_{\mathrm{p}} = \dfrac{\lambda_{\mathrm{c}} Q}{2\pi c}$ is the photon lifetime which is determined from the cavity quality Q-factor and the cavity resonance wavelength, λ_{c} , where c is the speed of light.

It is important to note that the model of Eqs. (9)-(10) avoids the *ad-hoc* introduction of the Purcell factor and the spontaneous emission factor, β , directly into the rate equations. This makes the treatment more transparent since only the physical parameters of the nanocavity LED such as the cavity dimensions and emitter/cavity relative linewidths are employed in the model.

Simulation results of Fig. 3b and Fig 4a

For the fitting of the L-I characteristics, Fig. 3b, the photon density was converted to an output power $P = n_{\mathrm{ph}} V \eta h c / \tau_{\mathrm{p}} \lambda_{\mathrm{c}}$, where h is the Planck's constant, and the parameter η was fixed to a value 4×10^{-2} and accounts for the estimated waveguide coupling efficiency, the grating out-coupling efficiency and the setup collection efficiency, as discussed in the main manuscript. The numerical results take into account the values of the calculated mode volume for the cavities reported here. For an ideal nanocavity (vertical sidewalls and low loss metal), we calculated an effective mode volume of the confined mode of $V = 3.05 \times 10^{-2}\,\mu\mathrm{m}^3$ (corresponding to $V \approx 2.5 \times (\lambda_{\mathrm{c}} / 2n_{\mathrm{ra}})^3$ when normalized to one half cubic wavelength in the material). For the realistic case of a nanocavity with high loss metal and 2° sidewalls, we

calculated an effective mode volume of $V = 7.6\times10^{-2}$ µm^3, and this value was used in the numerical simulation results presented here. We note that for the case of our nanocavity, which is not sub-wavelength in all three directions, most of the mode is confined in the semiconductor, so that the kinetic energy in the metal was neglected in the calculations of the effective mode volume. In the case of metal-clad cavities that are sub-wavelength in all three directions, the kinetic energy can be included in the calculations of the effective mode volume, as thoroughly discussed e.g. in the work of Khurgin and Sun [9]. As described in Eqs. (9) and (10), the spontaneous emission rate into the cavity mode is reduced by a factor of $V_{a,eff}/V_a$ as a result of the spatial dependence of the electric field in the active region in our metal nanocavity. For the ideal case of a nanocavity with vertical sidewalls, as represented in the mode profile of Fig. 1c, we calculate $V_{a,eff} = 1.77\times10^{-2}$ µm^3 resulting in $V_{a,eff}/V_a$~0.48. For the realistic case this value is reduced to ~0.35 ($V_{a,eff} = 1.3\times10^{-2}$ µm^3), and this value was used in the numerical simulations. Alternatives to our design that could make substantial improvements in the emitter-cavity overlap include developments of ultrathin III-V active layers in buried heterostructures [11] or nanoscale metallic coaxial devices composed by a metallic rod surrounded by a metal-coated semiconductor ring [12].

In the fitting of the L-I characteristic at low temperature in Fig. 3b, we employed typical values of InGaAs active material matched with InP found in the literature (values employed are described in the Supplementary Table 1), whereas the non-radiative recombination rate was neglected. The remaining parameters were taken from the experimental data. For the fitting of

the experimental results at room temperature (see L-Is in Fig. 3b), the term $r_{nr}(n)$ was included with C taken from the literature [13], the injection efficiency η_i was slightly adjusted and υ_s used as a fitting parameter. The other parameters (Supplementary Table 2) were either kept the same as for the low-temperature fitting (effective masses and quantum efficiencies), or taken from literature (energy bandgap), or from the experimental data (cavity frequency and quality factor). Finally, for the devices used in the modulation experiments in Fig 4a, the same procedure was used to fit the corresponding modulation properties and L-I characteristic at room temperature. The fitting parameters, $\upsilon_s = 2\times10^4$ cm s^{-1} and $\eta_i = 0.73$, provided the best agreement with the experimental data in that case.

Supplementary Table 1 | Material parameters for InGaAs and cavity at T=10 K

m_e^*	m_h^*	E_g (eV)	$\hbar\omega_{cav}$ (eV)	Q	η_i
$0.041\,m_0$	$0.45\,m_0$	0.82	0.83	62	0.8

Supplementary Table 2 | Material parameters for InGaAs and cavity at T=300 K

m_e^*	m_h^*	E_g (eV)	$\hbar\omega_{cav}$ (eV)	Q	η_i	υ_s (cm s^{-1})	C (cm^6 s^{-1})
$0.041\,m_0$	$0.45\,m_0$	0.752	0.8	37	0.75	4.5×10^4	8.5×10^{-29}

Simulation results and discussion of efficiency and modulation speed

We performed a systematic study to investigate the effects of the reduction of the surface recombination and the influence of the emitter-cavity spatial overlap and the reduction of the mode volume of the cavity in the performance of the nano-LED in terms of efficiency and modulation speed. In the Supplementary Fig. 9 we summarize the results of our findings that can be described as follows.

First, by assuming a reduction of the surface recombination (we employed the value of surface velocity 500 cm/s achieved recently in our work using an improved passivation method, see [14]) we observe a substantial improvement of the predicted efficiency of the nano-LEDs. The results demonstrate the possibility of achieving up to 100-fold increase of the output power at low injection currents, as seen in the red dashed curve in Supplementary Fig. 9a. However, the output power starts to saturate at higher injection current levels (>20 µA) due to the Auger recombination effect. While this has an effect in the reduction of the output power, it enhances the 3 dB bandwidth to close to 1 GHz, well beyond the radiative recombination limit (red curve in Supplementary Fig. 9b.

Further improvements of the output power and speed without compromising the efficiency can be achieved assuming the case of a nanocavity with physical dimensions smaller than our current devices. This corresponds to the Purcell enhanced regime via reduction of the effective mode volume of the nanocavity. In order to model this, we assumed $V = 3\times10^{-3}$ µm^3, corresponding to a reduction of the mode volume of about ten times the design values for our

nano-LED device (corresponding to almost three-times reduction in the lateral dimension). This value is similar to the smallest mode volumes reported in the literature of comparable metal-dielectric nanocavity devices (see, e.g. [15]). Since in the situation considered at least in one direction the cavity is still larger than $\lambda_c / 2n_{ra}$, calculations of the relative fraction of magnetic energy show that more energy is stored in the magnetic field than in the motion of electrons in the metal, [9] and therefore the mode volume definition in Eq. (6) is still valid. We also assumed the ideal case of a perfect emitter-cavity spatial overlap resulting in $V_{a,eff} \approx V$, a surface velocity of 500 cm/s, and a realistic room temperature Auger coefficient value (see Supplementary Table 2). In this Purcell enhanced regime with improved surface passivation, on-chip output powers above 1 µW can be predicted (assuming η=1), as shown in the blue dashed-dot color curve in Supplementary Fig 9a. The modulation speed is also substantially improved without compromising the efficiency. As shown in the blue curve of Supplementary Fig. 9b, speeds higher than 1 GHz are expected for injection levels above 30 µA. This means that Purcell-enhanced metal cavity nano-LED devices with improved surface passivation can potentially operate at room temperature at ~1 Gb/s rate with on-chip optical power levels above 1 µW (corresponding to ~10^4 photons/bit) and with energy consumptions <20 fJ/bit (operation at 10 µA and assuming a voltage drop of 1.4 V for the case of improved ohmic contacts). If we operate the nano-LED slightly below 1 µW output power, that is, in the current range of 1-4 µA, the corresponding energy consumption is 8 to 10.2 fJ/bit, respectively, and therefore in the range required by on-chip optical interconnects (i.e. <10 fJ/bit). Although further improvements of the bandwidth and energy consumption per bit are predicted by further downscaling of the nano-

LED, reducing the dimensions of the nanopillars is technologically challenging without considering changes in the current design (alternative designs can be found in [11] and[12]). Practical issues include an increased difficulty to obtain straight sidewall angles due to a strong mask erosion in the high aspect ratio hardmask needed to deeply etch pillars with small cross section (see Supplementary Fig. 3a). Additionally, the nanopillars might be under a large stress if the thickness of the metal cladding is significantly larger than the nanopillar diameter.

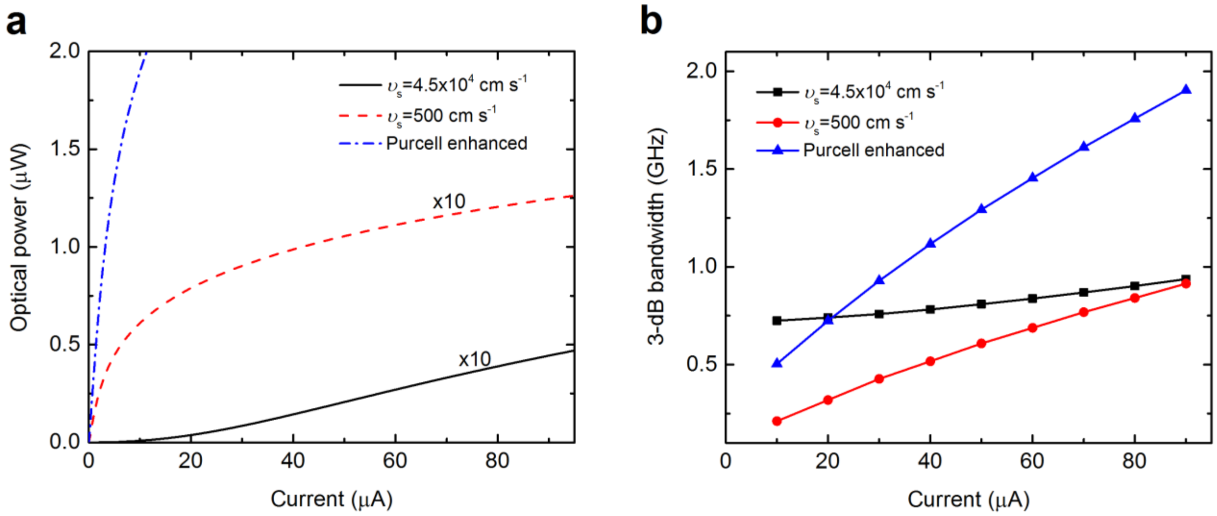

Supplementary Figure 9: Numerical simulation of electrically injected nano-LEDs. (a) L-I characteristics. The results show the numerical results of on-chip power levels, that is, using an external quantum efficiency of η=1. (b) Simulated 3-dB bandwidth vs injected current.

Supplementary Note 8: Nano-LED modulation speed: experimental results

Modulation speed at low-temperature

Supplementary Fig. 10a shows the time-resolved electroluminescence measurements at low-temperature (and corresponding model fit, Fig. 10b, neglecting non-radiative recombination effects) of the nano-LED devices presented in Fig. 4. The results show that at low-temperature the non-radiative losses can be considered negligible and the nano-LED modulation speed compares with the nanosecond-range radiative lifetime of the bulk intrinsic InGaAs active material.

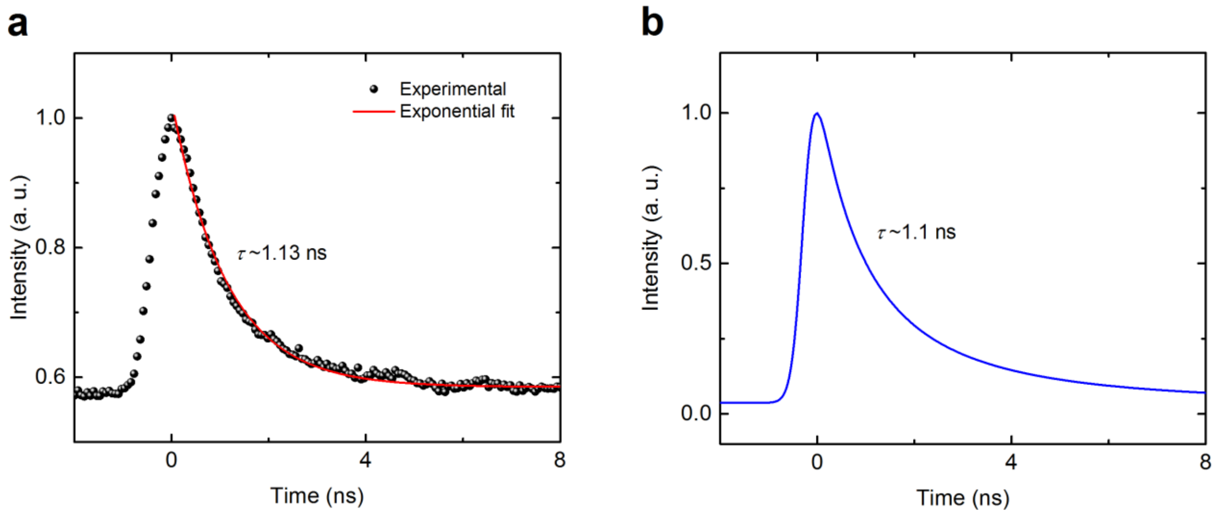

Supplementary Figure 10: Electro-optical modulation response of nanopillar LED. (a) Time-resolved electroluminescence showing the response of the device at 9.5 K to electrical pulses with widths of 500 ps and a bias current of 18.7 µA. Also shown is the nanosecond range recombination estimated from the fit with a single exponential decay curve. (b) Model fit.

High-speed switch-off beyond the limitation of the recombination rates

Further speed increase was achieved by operating our nano-LED under low bias to achieve a full turn-off cycle of the modulation enabling a fast sweep-out of carriers from the InGaAs active region in the nanopillars. Supplementary Fig. 11 shows a fast switch-off of around 123 ps at room temperature, a value beyond the limitation of the recombination rates. The exploitation of this ultrafast switch-off transient can already provide multi-GHz operation of the current nano-LEDs without requiring implementing other physical processes such as Purcell enhancement of the spontaneous emission rate. Although the switch-on time of our nanoLEDs is still limited by the carrier lifetimes, this can be reduced by using the current peaking techniques reported recently in [16] and[17]. Such type of techniques could potentially be implemented in our nanoLEDs using on-chip driving electronics hosted in the Si-substrate as enabled by the IMOS platform.

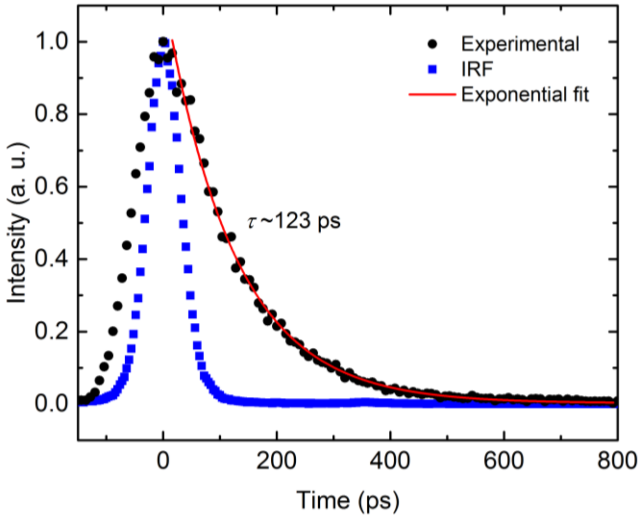

Supplementary Figure 11: Electro-optical response of nanopillar LED at low bias conditions. Time-resolved electroluminescence revealing ~123 ps switch-off of the nanopillar LED operating at room-temperature using modulation of electrical pulses with widths of 100 ps and a bias current of 3.13 µA. Also shown is the instrument response function (IRF) curve of our measurement setup.

Supplementary References

1. Bogaerts, W. *et al.* Fabrication of photonic crystals in silicon-on-insulator using 248-nm deep UV lithography. *IEEE J. Sel. Top. Quantum Electron.* **8,** 928–934 (2002).
2. Roelkens, G. *et al.* III-V/silicon photonics for on-chip and intra-chip optical interconnects. *Laser Photonics Rev.* **4,** 751–779 (2010).
3. Grigorescu, A. E. & Hagen, C. W. Resists for sub-20-nm electron beam lithography with a focus on HSQ: state of the art. *Nanotechnology* **20,** 292001 (2009).
4. van Delft, F. C. M. J. M., Weterings, J. P., van Langen-Suurling, A. K. & Romijn, H. Hydrogen silsesquioxane/novolak bilayer resist for high aspect ratio nanoscale electron-beam lithography. *J. Vac. Sci. Technol. B* **18,** 3419 (2000).
5. Lauvernier, D., Carette, M., Vilcot, J.-P., Bernard, D. & Decoster, D. Simple technological process for the fabrication of optical III-V nanowires integrated into a benzocyclobutene matrix. *ECS Trans.* **3,** 305–309 (2006).
6. Boroditsky, M. *et al.* Surface recombination measurements on III–V candidate materials for nanostructure light-emitting diodes. *J. Appl. Phys.* **87,** 3497–3504 (2000).
7. Yokoyama, H., Nambu, Y. & Kawakami, T. in *Confined Electrons and Photons: New Physics and Applications* (eds. Burstein, E. & Weisbuch, C.) 427–466 (Springer US, 1995). doi:10.1007/978-1-4615-1963-8_15
8. Gerry, C. & Knight, P. *Introductory Quantum Optics*. (Cambridge University Press, 2004).
9. Khurgin, J. B. & Sun, G. Comparative analysis of spasers, vertical-cavity surface-emitting lasers and surface-plasmon-emitting diodes. *Nat Phot.* **8,** 468–473 (2014).
10. Gregersen, N., Suhr, T., Lorke, M. & Mørk, J. Quantum-dot nano-cavity lasers with Purcell-enhanced stimulated emission. *Appl. Phys. Lett.* **100,** (2012).
11. Matsuo, S. *et al.* Directly modulated buried heterostructure DFB laser on SiO2/Si substrate fabricated by regrowth of InP using bonded active layer. *Opt. Express* **22,** 12139–12147 (2014).
12. Khajavikhan, M. *et al.* Thresholdless Nanoscale Coaxial Lasers. *Nature* **482,** 16 (2011).
13. Agrawal, G. P. & Dutta, N. K. *Semiconductor Lasers*. (Van Nostrand Reinhold, 1993).
14. Higuera Rodriguez, A. *et al.* Ultra-low surface recombination for deeply etched III-V semiconductor nano-cavity lasers. in *Advanced Photonics 2016 (IPR, NOMA, Sensors, Networks, SPPCom, SOF)* ITu2A.2 (Optical Society of America, 2016). doi:10.1364/IPRSN.2016.ITu2A.2
15. Hill, M. T. *et al.* Lasing in metallic-coated nanocavities. *Nat. Photonics* **1,** 589–594

(2007).

16. Halbritter, H., Jäger, C., Weber, R., Schwind, M. & Möllmer, F. High-Speed LED Driver for ns-Pulse Switching of High-Current LEDs. *IEEE Photonics Technology Letters* **26,** 1871–1873 (2014).

17. Binh, P. H., Renucci, P., Truong, V. G. & Marie, X. Schottky-capacitance pulse-shaping circuit for high-speed light emitting diode operation. *Electron. Lett.* **48,** 721 (2012).
